# The impact of economic crises on social inequalities in health: what do we know so far?

**DOI:** 10.1186/1475-9276-13-52

**Published:** 2014-07-25

**Authors:** Amaia Bacigalupe, Antonio Escolar-Pujolar

**Affiliations:** 1 Department of Sociology 2, University of the Basque Country (UPV/EHU), Leioa, Spain; 2 Local Health Authority, Cadiz, Spain

**Keywords:** Economic crisis, Health inequalities

## Abstract

Since 2008, Western countries are going through a deep economic crisis whose health impacts seem to be fundamentally counter-cyclical: when economic conditions worsen, so does health, and mortality tends to rise. While a growing number of studies have presented evidence on the effect of crises on the average population health, a largely neglected aspect of research is the impact of crises and the related political responses on social inequalities in health, even if the negative consequences of the crises are primarily borne by the most disadvantaged populations. This commentary will reflect on the results of the studies that have analyzed the effect of economic crises on social inequalities in health up to 2013. With some exceptions, the studies show an increase in health inequalities during crises, especially during the Southeast Asian and Japanese crises and the Soviet Union crisis, although it is not always evident for both sexes or all health or socioeconomic variables. In the Nordic countries during the nineties, a clear worsening of health equity did not occur. Results about the impacts of the current economic recession on health equity are still inconsistent. Some of the factors that could explain this variability in results are the role of welfare state policies, the diversity of time periods used in the analyses, the heterogeneity of socioeconomic and health variables considered, the changes in the socioeconomic profile of the groups under comparison in times of crises, and the type of measures used to analyze the magnitude of social inequalities in health. Social epidemiology should further collaborate with other disciplines to help produce more accurate and useful evidence about the relationship between crises and health equity.

## Introduction

Since 2008, Western countries are going through a deep crisis which has deeply penetrated into the economic, social, political and even ethical spheres of our societies. The high rates of unemployment, and increased poverty and social inequalities, particularly in southern Europe, are among its worst effects [1]. The neoliberal economic policies developed by most countries and imposed by the International Monetary Fund, the European Central Bank and the European Commission are hindering the process of decommodification –accessibility to public goods and services not provided by the market- that defined the model of welfare state in Europe during the last decades.

Evidence shows that the relationship between economic crises and health is fundamentally counter-cyclical: when economic conditions worsen, so do physical and mental health, and mortality tends to rise [2]. However, a pro-cyclical relationship has also been described, so that mortality increases in periods of expansion and decreases during economic downturns [3]. This could be due to improving health of people who remain in the labour market during recessions and the decline of some causes of death, which would compensate for the deterioration of health in other groups [4].

One aspect that has scarcely been researched so far is the impact of the economic crises on social inequalities in health [5]. At present it is clear that the negative consequences of the crisis are being primarily borne by the most disadvantaged populations, who are concentrating the major risks of some crucial social determinants of health, such as unemployment and poverty [6]. Moreover, the austerity policies are leaving these populations especially vulnerable to such disadvantages [7].

Many studies in recent years have shown that the unequal distribution of the social determinants of health undermines health equity [8]. However, the change in the pattern of social inequalities does not directly affect the profile of social inequalities in health within a population, since the interactions between both phenomena are complex [9,10]. The objective of this commentary is to reflect on the results of the peer-reviewed articles that have specifically analyzed the effect of economic crises on social inequalities in population health up to 2013. From their conclusions, we propose some ideas for a better understanding of what the effects of the current crisis and the austerity policies on health equity might be. Only studies that considered a pre-crisis period in their analyses and used some measure of socioeconomic position were included. On the contrary, the publications that did not clearly define which crisis they were referring to -since changes derived from more general economic restructuration processes or from economic stagnations were evaluated- were not included. The search was done in Pubmed and complemented with the revision of the references included in the retrieved articles.

## What are the consequences of crises on health inequalities?

The studies examined the effect of various economic crises during the nineties of the last century –the Soviet crisis, the financial crisis in Southeast Asia and Japan, and the crisis in the Nordic countries- as well as of the Great Recession beginning in 2008 (Table 1). They used diverse outcome variables -overall mortality, causes of death, and physical and mental health- and socioeconomic variables –mainly educational level, social class and employment status-. The periods analyzed were also heterogeneous, ranging from 4 [11,12] to 45 years [13]. Despite the existing evidence on the uneven effects of crises upon men and women [7], many studies did not stratify results by sex [11,12,14-17].

**Table 1:** Studies about the effect of economic crises on social inequalities in health

**Crisis**	**Country**	**Study period**	**Reference**	**Main health variable**	**Main socioeconomic variable**	**Analyses by sex**	**Main results**
**Great recession**	United Kingdom	2006-2010	Astell-Burt & Feng [12]	Limiting long-standing illness	Working status	No	Inequalities in limiting long-standing illness decreased from 2008
	United Kingdom	2004-2010	Harhay et al. [14]	Binge drinking	Working status; Income	No	The beginning of the crisis entailed an elevated risk of binge drinking among the unemployed. Income-related inequalities did not increase
	United Kingdom	1991-2010	Katikireddi et al. [32]	Mental health (GHQ-12)	Educational level; deprivation level	Yes	Inequalities in mental health were already increasing before 2008
	Spain	2006-2012	Rajmil et al. [15]	Obesity; Health behaviours; Mental health; Heath-related quality of life	Mothers’ educational level; Family’s working status	No	Inequalities in obesity among children did not change while inequalities in health-related quality of life increased
	Spain	2006-2010	Gili et al. [11]	Mental health	Educational level; Working status	No	Excepting the decrease of working status related inequalities in dysthymia, inequalities increased in all other mental health indicators
	Spain	2001-2011	Juárez et al. [27]	Perinatal health	Mothers’ educational level	Only women	Inequalities increased in macrosomia and in post-term births and did not increase in low birthweight and pre-term births
	Spain	2006-2012	Bartoll et al. [24]	Mental health (GHQ-12)	Social class; Educational level	Yes	Inequalities increased only in men
**Nordic crisis**	Finland	1981-1995	Valkonen et al. [20]	Total and cause-specific mortality	Social class	Yes	Inequalities were already increasing before the crisis, and continued growing afterwards
	Finland	1986-1994	Lahelma et al. [18]	Limiting long-standing illness; Self-perceived health	Educational level; Working status	Yes	Inequalities decreased in men (especially regarding limiting long-standing illness), and remained stable in women
	Finland	1993-2002	Lammintausta et al. [21]	Incidence and mortality for coronary disease	Income	Yes	Inequalities did not change during the period
	Sweden	1986-1995	Lundberg et al. [22]	Self-perceived health; Limiting long-standing illness	Social class; Educational level; Working status	Yes	Inequalities did not change during the period
	Nordic countries	1986-1995	Lahelma et al. [19]	Self-perceived health; Limiting long-standing illness	Working status; Educational level	Yes	Inequalities slightly decreased in men and did not change in women
**Soviet Union crisis**	Russia	1980-2001	Murphy et al. [28]	Life expectancy and mortality	Educational level	Yes	Inequalities in life expectancy increased especially among men since 1990
	Russia	1975-1998	Plavinski et al. [16]	Total and cause-specific mortality	Educational level	No	Inequalities increased during the 90s
**Southeast Asian and Japanese crises**	Japan	1955-2000	Fukuda et al. [13]	Life expectancy and mortality	Income	Yes	Absolute inequalities decreased from 1970 to 1995 while increased afterwards
	Japan	1986-2001	Kondo et al. [25]	Self-perceived health	Income; Social class	Yes	Inequalities did not change or slightly decreased in the population aged 40–60. In men, relative inequalities increased between middle-class non-manual and highest class workers
	Japan	1986-2007	Kachi et al. [23]	Self-perceived health	Income	Yes	After 1998, inequalities decreased because poor self-perceived health increased more among the highest income groups
	Korea	1993-2005	Son et al. [28]	Life expectancy and mortality	Educational level	Yes	Absolute inequalities in life expectancy at age 40 increased especially in men. Inequalities in mortality increased in the population aged 40-55
	Korea	1995-2006	Kim et al. [31]	Self-perceived health	Social class; Working status	Yes	Working status inequalities increased in all social classes. Among non-professionals, inequalities increased especially in women
	Korea	1998-2007	Hong et al. [17]	Depression; Suicidal behaviour	Income	No	Inequalities doubled in the period
	Korea	1989-2000	Khang et al. [26]	All-cause mortality; Self-perceived health	Educational level	Yes	In men, inequalities in mortality remained stable while inequalities in self-perceived health increased in both sexes
	Korea	1995-2005	Shim & Cho [30]	Alcohol-attributable mortality	Educational level; Social class	Only men	Inequalities increased in the period

With some exceptions [12,18-23], an increase in health inequalities during crisis periods is observed, although it is not always evident for both sexes [24-26] or for all health or socioeconomic variables [11,14,15,25-27]. The increase of inequalities in mortality was clear in Russia during the Soviet crisis [16,28], and in Korea and Japan during the Asian crises [13,29,30]. In Korea inequalities went up in mental health and self-perceived health [17,26,31], while less consistent results were observed in other studies of Japan [23,25]. Regarding the Great Recession, UK studies do not show an increase in health inequalities [12,32], with the exception of excessive alcohol consumption [14]. In Spain increased inequalities are observed in diagnosed mental pathology [11], health-related quality of life in children [15], perinatal health outcomes [27], and men’s mental health [24].

A clear exception occurred in the Nordic countries during the nineties, in which an increase in health inequalities was not observed. Indeed, inequalities in long-standing illness and self-perceived health decreased [18,19], and mortality did not increase more than it was already doing before the crisis in Finland [20]. Additionally, other studies show that inequalities in cardiovascular mortality, disability and self-perceived health in Sweden and Finland remained stable during the crisis [21,22].

## Why are these results observed? Reflections on the effect of the crises on health inequalities

According to the evidence on the impact of social determinants on health equity [8], increasing social inequality and poverty, greater job insecurity, rising unemployment, and privatization of public goods and services [7] can explain the increase in social inequalities in health during crises, as mainly described by the studies in Table 1.

However, some results also show that economic recessions are not always accompanied by an increase in social inequalities in health. Indeed, it seems that the role of welfare state policies during periods of recession is key to buffer the growth of inequalities, as happened in the Nordic countries during the crisis of the nineties [18]. Although there is no clear evidence that social inequalities in health are generally lower in the Nordic countries [33], it appears that their safety nets were effective in preventing the growth of social inequalities in health when economic conditions deteriorated. This probably prevented the most vulnerable groups from losing access to public goods and services, further social exclusion, and a decrease in their political advocacy capacity [9].

Additionally, there must be other factors explaining why in other countries where living conditions have deteriorated and austerity policies have been implemented in times of crisis, inequalities have not clearly risen [11,12,14,15,23-27]. Without being exhaustive, the following factors could partially explain this phenomenon:

Firstly, the diversity of time periods used may provide interesting clues: the use of short periods for the analyses-which is especially evident in the studies about the current crisis- makes it difficult to capture the long term effects on health inequalities. Indeed, it has been reported that the impact of unemployment and other adverse circumstances on health in times of crisis can be evident only after many years, especially in disadvantaged populations [34]. It is also very important to have a sufficient time-perspective prior to the beginning of the crisis in order to assess whether the crisis itself has had an added effect on trends that were already occurring. This perspective is usually absent in the studies analyzing the effects of the current economic crisis [11,12,15,24].

Secondly, we do not precisely know how deep the changes in the structural determinants of health must be in order to entail negative consequences in health for different social groups different social groups [35]. Nor is it clear in which health variables the effects of the crisis will be more rapidly noticeable [18]. It would seem logical that the effects on chronic health problems, on mortality from lung cancer -associated with an increase in smoking as a strategy for managing stress in crisis times-, or on disability shall be seen much later than other variables such as effects on self-perceived health or mental health [5].

Thirdly, the changes in the socioeconomic profile of the groups under comparison may also prevent from seeing an increase of inequalities in health. For example, in times of economic prosperity, the unemployed population is a small group of an especially disadvantaged socioeconomic profile, while in times of crisis, other less disadvantaged social groups can also become unemployed, which would weaken the usual relationship between unemployment and ill-health [36], at least among men [18]. This artificial phenomenon could consequently avoid that an increase in health inequalities among the employed and unemployed is seen during recessions [12,18,19]. However, this would not happen with other socioeconomic status variables such as educational level whose categories do not undergo rapid transformations by effect of the crisis.

Finally, the choice of measures to analyze the magnitude of inequalities and the changes may also influence the observed results. The exclusive use of effect measures in most of the studies -prevalence differences and ratios, or odds ratios- can omit a fundamental aspect: even if the strength of the relationship between socioeconomic status and health does not vary [21,22] or even decreases [12], inequalities may instead be growing because the weight of the population facing the risk factors (i.e. unemployment, poverty, etc.) is in fact increasing. Therefore, measures of total population impact should be used complementarily, so that the changes in the distribution of the population along the socioeconomic variable can be considered, and the effect of the crisis on health equity better assessed. However, only some of the studies include these kind of measures [13,24-26,30,32].

## Conclusions

This commentary has reviewed the studies that have examined so far the effects of the economic crises on social inequalities in health. Despite its wide methodological and approach variability, the negative impact of crises on health equity is shown, although not systematically. Additionally, some of the factors that can help shed some light on understanding their results have been described. However, the simultaneous occurrence of social phenomena in periods of pre-crisis and crisis both at the state and supra-national level make any understanding of the health impacts very complex.

Similarly as it happened with the debate about the impact of the crisis on the population’s health as a whole, it is likely that as the number of studies on the influence of the crisis on health equity increases, the scientific discourse will merge with the ideological one. The reason is that beyond what data indicate objectively, what is at stake is a much deeper debate about whether the neoliberal orientation of capitalism is exacerbating inequalities in health. In other words: which should be the best social and economical model for health equity?

To respond to this question, it will be convenient to incorporate new methodological approaches beyond hard quantification of impacts, which enables a better understanding of the negative consequences of current socioeconomic transformations for health and health equity. Moreover, we should not only be aware of the impacts of the current crisis, but also of those that will stem from the new social structure that is being progressively imposed by a minority [37] with the excuse of overcoming the crisis [38]. Therefore, today more than ever, epidemiology should enhance its social scope and work together with other disciplines to become more engaged in producing societies in which health equity is a central aim.
